# Testicular cancer and HPV semen infection

**DOI:** 10.3389/fendo.2012.00172

**Published:** 2012-12-21

**Authors:** Andrea Garolla, Damiano Pizzol, Alessandro Bertoldo, Marco Ghezzi, Umberto Carraro, Alberto Ferlin, Carlo Foresta

**Affiliations:** Department of Molecular Medicine, Section of Clinical Pathology, Centre for Human Reproduction Pathology, University of PadovaPadova, Italy

**Keywords:** chemotherapy, human papillomavirus, male infertility, radiotherapy, sexual transmitted diseases, sperm infection, sperm parameters, testicular cancer

## Abstract

Testicular cancer represents the more frequent solid tumor affecting males aged 15–35 years. In the last decades, its incidence showed a progressive increased probably due to genetic and environmental factors. Despite exposure to some viruses such as HIV, HCV, EBV, and HPV is frequently related to cancer development, there are no studies aimed to evaluate the possible implication of viral infections in the pathogenesis of testicular cancer. In this study, we analyzed sperm parameters and prevalence of HPV on sperm in 155 testicular cancer patients at diagnosis (T−1), after orchiectomy (T0) and after 12 months from surgery or from the end of adjuvant treatments (T12). All patients showed a significantly higher prevalence of semen infection than controls (9.5% and 2.4% respectively,) and altered sperm parameters both at T−1 and T0. Considering sperm parameters, at T−1 we observed a reduction of progressive motility, and after orchiectomy patients showed a reduction of sperm concentration and count and a further worsening of motility. Thereafter, patients were assigned to three groups on the basis of medical option after surgery: S = surveillance, R = radiotherapy, and C = chemotherapy +/− radiotherapy. At T12, untreated patients had an improvement of sperm parameters while R group and even more C group had a strong decrease of sperm number (*p* < 0.01 both vs. T0 and S group). Moreover, patients who received radio and/or chemotherapy had a very high prevalence of HPV semen infection (S = 7.7%, R = 30.8%, and C = 61.5%). In conclusion, patients with testicular cancer had frequently altered sperm parameters and higher prevalence of HPV semen infection that were worsened after radio and chemotherapy. Because HPV infection is a risk factor for cancer development and it may further reduce fertility, we suggest screening for HPV in testicular cancer patients at diagnosis and particularly after adjuvant treatments.

## Introduction

Testicular cancer represents a rare pathology, accounting for about 2% of total cancer. However, considering men aged 15–35, it becomes the most common solid malignancy (Siegel et al., 2012; Viatori, 2012). Moreover, many authors demonstrated an increasing secular trend especially in some regions of Europe and North America (Adami et al., 1994; Richiardi et al., 2004; Zoltick, 2011). Many risk factors have been studied as a pre-disposing factor in the development of this cancer, but only for some there is a high level of evidence (Senturia, 1987; Buetow, 1995). Conditions particularly involved are cryptorchidism, previous contralateral testicular cancer, family history and the presence of gonadal dysgenesis (Dieckmann and Pichlmeier, 2004; McGlynn and Trabert, 2012). Although many genital cancers are closely related to Papillomavirus (HPV), to date no study evaluated the possible role of viruses in the pathogenesis of testicular cancer. HPV is one of the most common sexually transmitted infections (STIs), it is particularly common in young sexually active population and its prevalence is closely related to sexual behavior (Dillner et al., 2000). HPV was found, from a long time, in a high percentage of genital benign lesions as warts and condylomata and carcinomas (Gissmann, 1984; Shah and Buscema, 1988). In particular is well-established the etiologic role of high-risk HPV types in the carcinogenesis of the vulva, vagina, penis, anus, head, neck, and oropharyngeal cavity (Backes et al., 2009; Smith et al., 2009; Guily et al., 2011). Recently, we reported a significant presence of (HPV) DNA in thawed semen samples from patients with testicular cancer who cryopreserved semen (Foresta et al., 2010). This finding was confirmed by other authors showing similar data from patients who banked their sperm because of chemo/radiotherapy (Kaspersen et al., 2011). These data are very important because cryopreserved semen samples, especially when patients undergo therapies that can affect spermatogenesis, are frequently used by assisted reproductive techniques, such as intra-cytoplasmic sperm injection (ICSI). It has been observed that the bond between virus and sperm is very strong and that conventional washing procedures aimed to select sperm before *in vitro* fertilization (IVF) have low efficacy in eliminating HPV sperm infection (Foresta et al., 2011a; Garolla et al., 2012). In addition, we demonstrated that artificially infected sperm, both transfected with E6/E7 HPV genes and incubated with viral proteins, are able to enter the oocyte, to deliver HPV genome, and that viral genes are then actively transcribed by the penetrated oocyte (Foresta et al., 2011b). Furthermore, a recent study showed that HPV infected couples undergoing assisted reproduction techniques (ART) cycles experienced an increased risk of pregnancy loss compared to non-infected ones (Perino et al., 2011). In these couples the more predictive factor of early abortion was represented by HPV sperm infection. An important risk factor for HPV infection is the presence of immunosuppression status. Patients with cancer, particularly after adjuvant therapies, as chemotherapy and radiotherapy can be immunosuppressed and therefore at higher risk for this infection (Rasmussen and Arvin, 1982; Bieber et al., 2006; Fallahian et al., 2010). Despite the large body of literature concerning the role of HPV in many cancer types, very little is known about this viral infection and testicular cancer. In the light of these considerations, we evaluated semen parameters and the prevalence of HPV semen infection in a large group of patients with testicular cancer at diagnosis, after orchiectomy and after further 12 months of surveillance or after the end of radiotherapy and/or chemotherapy. In subjects with HPV semen infection, we evaluated also the percentage of infected sperm.

## Materials and methods

### Patients

Written informed consent was obtained from all patients, and the study protocol was approved by the local ethics committee. We enrolled 155 consecutive patients affected by testicular cancer candidate to unilateral orchiectomy, who attended the Centre for Human Reproduction Pathology for andrological examination. A medical history including previous circumcision, smoking, and sexual behavior was obtained from each patient. As control subjects, we used a group of 84 healthy proven fertile men. All subjects collected semen for standard semen analysis, detection of HPV DNA and fluorescence *in situ* hybridization (FISH) for HPV at diagnosis and 1 month after orchiectomy. Therefore, patients who underwent chemo and/or radiotherapy were reevaluated after 12 months from the end of treatments. Patients who were followed-up just by surveillance repeated the same analysis after 12 months from surgery.

### Semen processing

Semen samples were obtained by masturbation after 3 days of sexual abstinence. After liquefaction at room temperature, semen volume, pH, sperm concentration, viability, motility, and normal morphology were determined following World Health Organization guidelines for semen analysis (WHO, 2010). An aliquot was used for further analysis.

### HPV DNA detection

The presence of HPV infection was detected by PCR amplification of HPV DNA with SPF10 primers and then genotyped by the INNO-LiPA HPV Genotyping Extra assay (Innogenetics, Gent, Belgium), which can identify the following HPV genotypes: HPV-6, 11, 16, 18, 26, 31, 33, 35, 39, 40, 42, 43, 44, 45, 51, 52, 53, 54, 56, 58, 59, 66, 68, 69/71, 70, 73, 74, and 82 (Eklund et al., 2010).

### Sperm fluorescence *in situ* hybridization (FISH) for HPV

Samples containing at least 2 × 10^6^ ejaculated sperm were fixed in a methanol-acetic acid solution for at least 1 h at −20°C. To permeabilize, samples were digested with pepsin diluted 1:25,000 in pre-warmed 0.01 mol/L-1 HCl for 10 min at 37°C. Permeabilization of the specimens was stopped with 3–5-min washes in PBS 1×; then samples were dehydrated in 70%, 80%, and absolute ethanol for 2 min and finally air-dried. Samples were then overlaid with 20 mL of hybridization solution (Pan Path, Amsterdam), containing biotin-labeled HPV DNA probe (a mix of total genomes containing the conserved HPV region). Each slide was covered with a glass coverslip, and the edges were sealed with nail polish to prevent loss of the mixture during denaturation and hybridization. After a simultaneous denaturation of cellular target DNA and HPV DNA probe on a heating block for 5 min at 95°C, hybridization was performed by incubating the samples at 37°C overnight in a humidified chamber. Thereafter, the coverslips were carefully removed and the slides were washed in PBS 1× for 10 min. After 15 min incubation at 37°C with the differentiation reagent (Pan Path), the slides were washed three times in PBS 1×. The biotin-labeled HPV probe was detected by incubation with 1:200 streptavidin Texas Red® (Vector Laboratories, Burlingame, CA) for 40 min at room temperature. After detection, the slides were washed twice in PBS 1×/0.01% Triton and then twice in PBS 1× and mounted with a solution containing 4′, 6-diamidino-2phenylindole (DAPI) and anti-fade (BioBlue, BioView Ltd., Nes Ziona, Israel). Samples were analyzed using a fluorescence microscope (Nikon ViCo Video Confocal Microscope) equipped with a triple bandpass filter set (FITC, TRITC, DAPI). For each slide, at least 200 spermatozoa were analyzed. Evaluation of nuclear hybridization signals was performed in triplicate by different investigators. In samples that resulted negative during the follow-up, results were confirmed by PCR. When nuclei were completely and homogeneously stained and multiple small spots or single large signals were present, the sperm cells were classified as positive. The method was tested on control slides containing CaSki cells, a human cervical carcinoma cell line with stably integrated and transcriptionally active HPV genomes, which served as a control for the specific probe. Cells smeared on salinated glass slides were fixed with 4% paraformaldehyde in PBS for 10 min. After fixation, cells were subjected to 3–5-min washes in PBS 1× and then dehydrated with 5-min ethanol washes (30%, 60%, and 95%). Cell smears were then air-dried and stored at 4°C until use.

### Statistical analysis

The values shown are the averages of at least three evaluations performed by different operators. Data regarding sperm parameters were presented as mean ± SD while data on semen infection as percentage. Differences between data were determined by two-tailed Student's *t*-test after acceptance of normal distribution with the Kolmogorov-Smirnov test. Comparisons of proportion were performed with a one-sided non-parametric resampling test. *P*-values (two sided) <0.05 were considered statistically significant.

## Results

Mean age of patients with testicular cancer was 31.2 ± 5.4 years, not different from control subjects (30.8 ± 4.7 years). In Table 1 are reported sperm parameters and HPV DNA detection in semen samples from controls and from patients evaluated before (T−1) and 1 month after orchiectomy (T0). At T−1, patients showed sperm concentration, count, and morphology not different from controls, but a significant reduction of progressive motility (*p* < 0.05). After orchiectomy, we found a significant reduction of sperm concentration and count (both *p* < 0.05 vs. T−1) and a further reduction of sperm motility compared to T−1 and control subjects (*p* < 0.01). Moreover, sperm volume and morphology were not significantly different in patients compared with controls before and after orchiectomy. Interestingly, the percentage of subjects with HPV infection was higher among patients than controls (9.7% vs. 2.4%). This percentage remained unchanged at T0. After orchiectomy, patients were assigned to three groups based on medical option: S = candidate to surveillance, R = candidate to radiotherapy, and C = candidate to chemotherapy with or without radiotherapy. In Table 2, are reported sperm parameters, the percentage of patients with semen infection and percentage of infected sperm observed in the three groups at T0 and after 12 months of surveillance or from the end of radio and or chemotherapy (T12). At T0, considering patients from different groups we found no difference in sperm parameters and prevalence of HPV infection. Six patients were lost during follow-up (4 from group S, 1 from group R, and 1 from group C), therefore data at T12 are referred to the remaining 149 patients. Considering patients as a whole, the mean values of sperm parameters observed at T12 was not different from that at T0. However, patients who underwent surveillance had a significant increase of sperm number and motility compared to T0 (*p* < 0.05). In contrast, subjects who had radiotherapy showed no increase in sperm number and reduced sperm motility compared to S group (*p* < 0.05). Moreover, patients who underwent chemotherapy associated or not with radiotherapy, had a reduction of both sperm number and motility vs. T0 vs. patients from groups S (*p* < 0.05 and *p* < 0.01 respectively). In the three groups, we found no significant differences in both ejaculate volume and normal morphology comparing T12 with T−1 and T0. Considering all patients, HPV semen infection was significantly higher in patients at T12 respect to T0 (34.9% vs. 9.7%). By the comparison of different groups, the prevalence of infection resulted 7.7%, 30.8% and 61.5% in S, R, and C groups respectively. Finally, patients treated by chemotherapy had also a higher percentage of HPV infected sperm compared to T0 and to S (both *p* < 0.05). In Figure 1 there is an example of FISH analysis for HPV performed in a semen sample from testicular cancer patients.

**Table 1:** **Sperm parameters and HPV DNA detection in semen samples from control subjects and patients at the time of diagnosis (T−1) and 1 month after orchiectomy (T0)**.

	**Ejaculate volume (mL)**	**Sperm concentration (million/mL)**	**Sperm count (million)**	**Progressive motility (%)**	**Normal morphology (%)**	**Positive PCR (%)**
Controls (*n* = 84)	3.3 ±0.8	46.9 ± 11.3	153.0 ± 53.1	53.4 ± 12.1	21.6 ± 6.4	2 (2.4)
T−1 (*n* = 155)	3.2 ± 1.3	40.1 ± 45.2	110.1 ± 125.3	34.2 ± 17.7^*^	18.9 ± 8.3	15 (9.7)^*^
T0 (*n* = 155)	3.1 ± 1.3	19.8 ± 16.6^**^^,^ ^#^	61.9 ± 45.5^**^^,^ ^#^	31.2 ± 15.1^**^	18.5 ± 9.3	15 (9.7)^*^

* p < 0.05 vs. control subjects.

** p < 0.01 vs. control subjects.

# p < 0.05 vs. T−1.

**Table 2:** **Sperm parameters, HPV DNA detection and FISH for HPV observed in testicular cancer patients 1 month after orchiectomy (T0) and after 12 months (T12) from the end of any treatment**.

**Groups**	**Ejaculate volume (mL)**	**Sperm concentration (million/mL)**	**Sperm count (million)**	**Progressive motility (%)**	**Normal morphology (%)**	**Positive PCR (%)**	**FISH on sperm (%)**
T0							
S (*n* = 46)	2.9 ± 1.3	19.4 ± 14.4	54.6 ± 45.2	30.9 ± 14.1	19.8 ± 9.5	5 (11.1)	22.4 ± 8.3
R (*n* = 55)	3.2 ± 1.5	20.7 ± 19.7	67.1 ± 55.7	33.7 ± 17.6	17.9 ± 9.2	4 (7.2)	24.0 ± 3.7
C (*n* =54)	3.1 ± 1.1	19.4 ± 15.2	62.7 ± 60.2	29.1 ± 12.9	17.9 ± 9.3	6 (10.9)	25.3 ± 3.8
Total (*n* = 155)	3.1 ± 1.3	19.8 ± 16.6	61.9 ± 45.5	31.2 ± 15.1	18.5 ± 9.3	15 (9.7)	24.0 ± 5.4
T12							
S (*n* = 42)	3.1 ± 1.5	35.5 ± 25.7^*^	94.2 ± 93.5^*^	43.7 ± 18.2^*^	18.6 ± 10.1	4 (7.7)	24.9 ± 3.7
R (*n* = 54)	3.1 ± 1.7	23.8 ± 20.6	69.9 ± 58.7	30.8 ± 15.3^#^	18.0 ± 13.7	16 (30.8)^*^^,^ ^#^	28.7 ± 6.0
C (*n* = 53)	3.3 ± 1.5	11.1 ± 9.9^*^^,^ ^##^	24.4 ± 17.4^*^^,^ ^##^	20.8 ± 14.2^*^^,^ ^##^	17.1 ± 11.2	32 (61.5)^**^^,^ ^##^	39.6 ± 8.2^*^^,^ ^#^
Total (*n* = 149)	3.2 ± 1.5	22.6 ± 20.6	61.3 ± 50.5	30.7 ± 18.5	17.8 ± 11.5	52 (34.9)^**^	29.4 ±6.9

Data are shown as total and subgrouping patients in: S group = patients who underwent surveillance, R group = patients who underwent radiotherapy, and C group = patients who underwent chemotherapy followed or not by radiotherapy.

* p < 0.05 vs. T0.

** p < 0.01 vs. T0.

# p < 0.05 vs. S.

## p < 0.01 vs. S.

**Figure 1:**
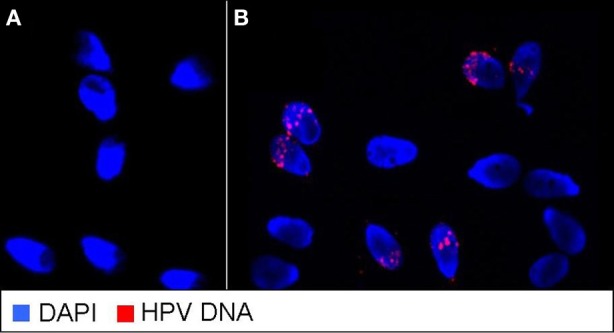
**An example of FISH analysis for HPV performed on infected semen samples from patients with testicular cancer.** Red staining indicates the presence of HPV DNA. **(A)** Negative semen sample. **(B)** Infected semen sample.

## Discussion

It is of concern, especially during last years, the increasing incidence of reproductive tract diseases and particularly testicular cancer (Richardson et al., 2012). The latter disease is particularly found among males aged between 15 and 40 years (Siegel et al., 2012; Viatori, 2012) and, although it is a curable disease with a good prognosis, it is a major risk factor for fertility (Jacobs and Vaughn, 2012). In fact, patients with testicular cancer have frequently a concomitant alteration of spermatogenesis already at diagnosis, that can be further affected by chemo or radiation therapies (Schrader et al., 2001; Bieber et al., 2006). In this paper, we considered sperm parameters and prevalence of HPV semen infection in testicular cancer patients pre- and post-orchiectomy. Moreover, after 12 months we evaluated the effect of different medical options on the same parameters. As previously reported (Selice et al., 2011), in affected patients we observed reduced sperm motility at diagnosis that was still present 1 month after orchiectomy together with a significant reduction of sperm number. Interestingly, we found that patients at diagnosis had a significant increase of HPV semen infection compared to controls. This finding could represent a cause of testicular cancer or an effect of this malignancy. Many hypotheses have been raised regarding the possible cause of testicular cancer development, as in utero and pre-pubertal exposure to endocrine didisrupters, genetic predisposition, and environmental conditions. Despite it is well-recognized that some viral infection have a major role in the development of many cancers (EBV, HCV, HPV, HIV etc.), this link has never been proposed for testicular tumors. On the other hand all tumors, including testicular cancer, may induce an impairment of general health exposing patients to infections. Moreover, testicular cancer patients after orchiectomy may undergo to different medical options based on type and tumor staging. Low risk conditions frequently allow just surveillance, while conditions at higher risk are usually treated by adjuvant therapies. Patients from S group had a significant improvement of sperm number and motility. This finding suggests that the residual testis may have a compensatory role after contralateral orchiectomy if patients are not exposed to treatments affecting spermatogenesis. Far from many years, it is well-known that both chemo and radiotherapies deeply impair spermatogenesis and immune system. The results of this study demonstrated that at T12 patients who received radio and even more those who received chemotherapy had altered sperm parameters both vs. pre-treatment vs. surveillance group. The strong association between kind of treatment and sperm alteration is further underlined by the comparison of sperm parameters from different groups at T0. This analysis showed no difference, suggesting that there was no relation between sperm parameters and the degree of malignancy at diagnosis. In the same way, adjuvant treatments resulted strongly related to HPV infection susceptibility. In this study, we found that after surveillance 7% of patients had HPV semen infection rising to 31% and 61% after radio and chemotherapy respectively. Therefore, drug-mediated immunosuppression seems to be strictly related to HPV seminal infection in testicular cancer patients. Finally considering FISH analysis for HPV, beside a trend to increase in R group, we observed a significantly higher percentage of infected sperm in subjects who received chemotherapy. This is the first study evaluating HPV sperm infection in semen samples from patients with testicular cancer. By our findings we can conclude that HPV infection is more prevalent in affected patients than controls. However, as recently suggested by other authors (Bertazzoni et al., 2013), this infection seems not to be cause of testicular cancer, but rather the effect of a status of higher vulnerability induced by the tumor. More and larger studies of follow-up of HPV infected patients will clarify whether or not there is a cause-effect relationship between HPV and testicular cancer or *vice versa*. Moreover, our results demonstrated that treatments frequently used in testicular cancer patients and in particular chemotherapy, are able to strongly increase the prevalence of HPV semen infection, probably through their immunosuppressive action. In conclusion, we suggest testing sperm HPV in patients affected by testicular cancer before and particularly after treatments inducing immunosuppression, because this infection can in turn induce cancer in many sites and further reduce male fertility. Further and larger studies are needed to confirm our findings also in patients affected by other tumors.

### Conflict of interest statement

The research was conducted in the absence of any commercial or financial relationships that could be construed as a potential conflict of interest.

## References

[B1] Adami H. O. Bergström R. Möhner M. Zatoñski W. Storm H. Ekbom A. (1994). Testicular cancer in nine northern European countries. Int. J. Cancer 59, 33–38 7927900 10.1002/ijc.2910590108

[B2] Backes D. M. Kurman R. J. Pimenta J. M. Smith J. S. (2009). Systematic review of human papillomavirus prevalence in invasive penile cancer. Cancer Causes Control 20, 449–457 10.1007/s10552-008-9276-9 19082746

[B3] Bertazzoni G. Sgambato A. Migaldi M. Grottola A. Sabbatini A. M. Nanni N. (2013). Lack of evidence for an association between seminoma and human papillomavirus infection using GP5+/GP6+ consensus primers. J. Med. Virol. 85, 105–109 10.1002/jmv.23431 23073996

[B4] Bieber A. M. Marcon L. Hales B. F. Robaire B. (2006). Effects of chemotherapeutic agents for testicular cancer on the male rat reproductive system, spermatozoa, and fertility. J. Androl. 27, 189–200 10.2164/jandrol.05103 16278370

[B5] Buetow S. A. (1995). Epidemiology of testicular cancer. Epidemiol. Rev. 17, 433–449 8654520 10.1093/oxfordjournals.epirev.a036202

[B6] Dieckmann K. P. Pichlmeier U. (2004). Clinical epidemiology of testicular germ cell tumors. World J. Urol. 22, 2–14 10.2164/jandrol.05103 15034740

[B7] Dillner J. Meijer C. J. von Krogh G. Horenblas S. (2000). Epidemiology of human papillomavirus infection. Scand. J. Urol. Nephrol. Suppl. 205, 194–200 11144898

[B8] Eklund C. Zhou T. Dillner J. WHO Human Papillomavirus Laboratory Network. (2010). Global proficiency study of human papillomavirus genotyping. J. Clin. Microbiol. 48, 4147–4155 10.1128/JCM.00918-10 20844222 PMC3020877

[B9] Fallahian F. Alavian S. M. Fallahian V. Zamani F. (2010). Impact of immunosuppression and chemotherapy on reactivation of viral hepatitis. Saudi J. Kidney Dis. Transpl. 21, 621–627 20587863

[B10] Foresta C. Ferlin A. Bertoldo A. Patassini C. Zuccarello D. Garolla A. (2010). Human papilloma virus in the sperm cryobank: an emerging problem? Int. J. Androl. 34, 242–246 10.1111/j.1365-2605.2010.01075.x 20522126

[B12] Foresta C. Pizzol D. Bertoldo A. Menegazzo M. Barzon L. Garolla A. (2011a). Semen washing procedures do not eliminate human papilloma virus sperm infection in infertile patients. Fertil. Steril. 96, 1077–1082 10.1016/j.fertnstert.2011.04.009 21536283

[B13] Foresta C. Patassini C. Bertoldo A. Menegazzo M. Francavilla F. Barzon L. (2011b). Mechanism of human papillomavirus binding to human spermatozoa and fertilizing ability of infected spermatozoa. PLoS ONE 6:e15036 10.1371/journal.pone.0015036 21408100 PMC3051064

[B14] Garolla A. Lenzi A. Palù G. Pizzol D. Bertoldo A. De Toni L. (2012). Human papillomavirus sperm infection and assisted reproduction: a dangerous hazard with a possible safe solution. Hum. Reprod. 27, 967–973 10.1093/humrep/des009 22313870

[B15] Gissmann L. (1984). Human papillomavirus DNA in genital tumours. IARC Sci. Publ. 63, 405–411 6100278

[B16] Guily J. L. Jacquard A. C. Prétet J. L. Haesebaert J. Beby-Defaux A. Clavel C. (2011). Human papillomavirus genotype distribution in oropharynx and oral cavity cancer in France–The EDiTH VI study. J. Clin. Virol. 51, 100–104 10.1016/j.jcv.2011.03.003 21527208

[B17] Jacobs L. A. Vaughn D. J. (2012). Hypogonadism and infertility in testicular cancer survivors. J. Natl. Compr. Canc. Netw. 10, 558–563 22491052 10.6004/jnccn.2012.0053

[B18] Kaspersen M. D. Larsen P. B. Ingerslev H. J. Fedder J. Petersen G. B. Bonde J. (2011). Identification of multiple HPV types on spermatozoa from human sperm donors. PLoS ONE 6:e18095 10.1371/journal.pone.0018095 21479232 PMC3066218

[B19] McGlynn K. A. Trabert B. (2012). Adolescent and adult risk factors for testicular cancer. Nat. Rev. Urol. 9, 339–349 10.1038/nrurol.2012.61 22508459 PMC4031676

[B20] Perino A. Giovannelli L. Schillaci R. Ruvolo G. Fiorentino F. P. Alimondi P. (2011). Human papillomavirus infection in couples undergoing *in vitro* fertilization procedures: impact on reproductive outcomes. Fertil. Steril. 95, 1845–1848 10.1016/j.fertnstert.2010.11.047 21167483

[B21] Rasmussen L. Arvin A. (1982). Chemotherapy-induced immunosuppression. Environ. Health Perspect. 43, 21–25 7037385 10.1289/ehp.824321 PMC1568884

[B22] Richardson L. C. Neri A. J. Tai E. Glenn J. D. (2012). Testicular cancer: a narrative review of the role of socioeconomic position from risk to survivorship. Urol. Oncol. 30, 95–101 10.1016/j.urolonc.2011.09.010 22127018 PMC4698969

[B23] Richiardi L. Bellocco R. Adami H. O. Torrång A. Barlow L. Hakulinen T. (2004). Testicular cancer incidence in eight northern European countries: secular and recent trends. Cancer Epidemiol. Biomarkers Prev. 13, 2157–2166 15598775

[B24] Schrader M. Heicappell R. Müller M. Straub B. Miller K. (2001). Impact of chemotherapy on male fertility. Onkologie 24, 326–330 11574759 10.1159/000055103

[B25] Selice R. Ferlin A. Garolla A. Caretta N. Foresta C. (2011). Effects of endogenous FSH on normal human spermatogenesis in adults. Int. J. Androl. 34, e511–e517 10.1111/j.1365-2605.2010.01134.x 21790654

[B26] Senturia Y. D. (1987). The epidemiology of testicular cancer. Br. J. Urol. 60, 285–291 3319010 10.1111/j.1464-410x.1987.tb04970.x

[B27] Shah K. V. Buscema J. (1988). Genital warts, papillomaviruses, and genital malignancies. Annu. Rev. Med. 39, 371–379 10.1146/annurev.me.39.020188.002103 2835929

[B28] Siegel R. Naishadham D. Jemal A. (2012). Cancer statistics, 2012. CA. Cancer J. Clin. 62, 10–29 22237781 10.3322/caac.20138

[B29] Smith J. S. Backes D. M. Hoots B. E. Kurman R. J. Pimenta J. M. (2009). Human papilloma virus type-distribution in vulvar and vaginal cancers and their associated precursors. Obstet. Gynecol. 113, 917–924 10.1097/AOG.0b013e31819bd6e0 19305339

[B30] Viatori M. (2012). Testicular cancer. Semin. Oncol. Nurs. 28, 180–189 10.1016/j.soncn.2012.05.007 22846486

[B31] World Health Organisation. (2010). WHO Laboratory Manual for the Examination and Processing of Human Semen, 5th Edn Geneva: World Health Organization

[B32] Zoltick B. H. (2011). Shedding light on testicular cancer. Nurse Pract. 36, 32–39 10.1097/01.NPR.0000398870.16580.86 21685776

